# INTERPROFESSIONAL GERIATRIC ASSESSMENT CLINIC: STUDENT SKILL DEVELOPMENT AND PATIENT SATISFACTION

**DOI:** 10.1093/geroni/igad104.3439

**Published:** 2023-12-21

**Authors:** Ryan Butler, Lisa Phifer, Katie Ehlman, Suzanne Leahy, Ashley Keller, Haneul Jung

**Affiliations:** University of Southern Indiana, Evansville, Indiana, United States; University of Southern Indiana, Evansville, Indiana, United States; University of Southern Indiana, Evansville, Indiana, United States; University of Southern Indiana, Evansville, Indiana, United States; University of Southern Indiana, Evansville, Indiana, United States; University of Southern Indiana, Evansville, Indiana, United States

## Abstract

The University of Southern Indiana Interprofessional Geriatric Assessment Clinic (IGAC) provides interprofessional clinical experience to medical residents and health profession students using the Institute for Healthcare Improvement’s Age-Friendly 4 Ms model while addressing the complex health conditions of medically underserved older adult patients. This study examines student skill development and patient satisfaction. A nursing student, medical resident, Area Agencies on Aging (AAA) case manager, and an occupational therapy or physical therapy student utilize evidence-based assessment tools to examine the four elements of Age-Friendly 4 Ms care, including high risk medications, mobility, mentation, and what matters. Students (N=56) completed a retrospective pre-posttest measuring six skills related to IGAC activities. In addition, patients (N=42) completed a posttest measuring satisfaction of the IGAC visit. “Administering standardized health assessments” and “using information about patient social needs to develop healthcare recommendations” were the two IGAC skills wherein students perceived the largest gains, based on paired samples t-tests of significance and Cohen’s d measurement of effect size. Students also reported statistically significant, moderately sized gains in “documenting patient measurements in a medical chart”, “communicating medical information to other health professionals”, and “conducting clinical visits with older adult patients”. Ninety-two percent of patients were satisfied with the overall actions of the care team for their visits. This research indicates that the USI IGAC both increases student skill development and supports patient satisfaction.

